# Triamcinolone Acetonide affects TGF-β signaling regulation of fibrosis in idiopathic carpal tunnel syndrome

**DOI:** 10.1186/s12891-018-2260-y

**Published:** 2018-09-22

**Authors:** Tai-Hua Yang, Anne Gingery, Andrew R. Thoreson, Dirk R. Larson, Chunfeng Zhao, Peter C. Amadio

**Affiliations:** 1 Biomechanics & Tendon and Soft Tissue Biology Laboratory, Division of Orthopedic Research, Rochester, USA; 2 Division of Biomedical Statistics and Informatics, Department of Health Science Research, Mayo Clinic, Rochester, MN 55905 USA; 3 Tendon and Soft Tissue Biology Laboratory, Division of Orthopedic Research, Mayo Clinic, 200 First Street SW, Rochester, MN 55905 USA

**Keywords:** Carpal tunnel syndrome, Collagen gel contraction, Fibrosis, Subsynovial connective tissue, Triamcinolone Acetonide

## Abstract

**Background:**

Fibroblast behavior and cell-matrix interactions of cells from normal and idiopathic carpal tunnel syndrome (CTS) subsynovial connective tissue (SSCT) with and without Triamcinolone Acetonide (TA) were compared in this study. A cell-seeded gel contraction model was applied to investigate the effect of steroid treatment on SSCT fibroblast gene expression and function.

**Methods:**

SSCT cells were obtained from CTS patients and fresh cadavers. Cells were isolated by mechanical and collagenase digestion. Collagen gels (1 mg/ml) were prepared with SSCT cells (1 × 10^6^/mL). A sterile Petri dish with a cloning ring in the center was prepared. The area between the ring and outer dish was filled with cell-seeded collagen solution and gelled for 1 h. The gel was released from the outer way of the petri dish to allow gel contraction. Cell seeded gels were treated with 10 M triamcinolone acetonide (TA) or vehicle (DMSO) in modified MEM. Every 4 h for 3 days the contracting gels were photographed and areas calculated. Duplicate contraction tests were performed with each specimen, and the averages were used in the analyses, which were conducted using two-factor analysis of variance in a generalized linear model framework utilizing generalized estimating equations (GEE) to account for the correlation between samples. The contraction rate was determined by the area change over time, and the decay time constant was calculated. A customized mechanical test system was used to determine gel stiffness and tensile strength. Gene expression was assessed using Human Fibrosis and Cell Motility PCR arrays.

**Results:**

TA-treated gels had a significantly higher contraction rate, tensile strength and stiffness than the untreated gels. Proteinases involved in remodeling had increased expression in TA-treated gels of the patient group. Pro-fibrotic genes and ECM regulators, such as TGF-β, collagens and integrins, were down-regulated by TA, indicating that TA may work in part by decreasing fibrotic gene expression.

**Conclusions:**

This study showed that TA affects cell-matrix interaction and suppresses fibrotic gene expression in the SSCT cells of CTS patients.

**Electronic supplementary material:**

The online version of this article (10.1186/s12891-018-2260-y) contains supplementary material, which is available to authorized users.

## Background

In the United States, hundreds of thousands of carpal tunnel release (CTR) procedures are performed each year [1, 2]. Although most cases of carpal tunnel syndrome (CTS) are idiopathic [3, 4], other causes have been reported, such as repetitive motion, congenital anomalies, autoimmune disorders, arthritis and trauma [5–7]. Subsynovial connective tissue (SSCT) fibrosis and vascular proliferation have been reported as significant factors in CTS development [8, 9]. This fibrosis impedes the normal ulnodorsal motion of the median nerve when the hand is pinching and gripping while the wrist is flexed and may therefore result in nerve compression between the flexor tendons and flexor retinaculum [10–12].

Surgical decompression is the most common treatment of moderate to severe CTS [13]. However, non-surgical treatments, such as anti-inflammatory medication, corticosteroid injection, immobilization and physical therapy, are often used in milder cases [14–16]. Steroid injection was first described by Kendrick in 1957 [17]. Subsequent studies have reported a wide range of success rates, from 50 to 90% at 4–12 weeks and less at longer time points [14, 15].

One concern about the effectiveness of corticosteroid therapy for CTS is that these drugs are normally used for their anti-inflammatory effect, whereas the fibrosis in CTS is typically non-inflammatory [8, 11]. Therefore, the mechanism by which local steroid injection affects CTS remains unknown.

In this study, our hypothesis was that steroid treatment reduces TGF-β–related fibrosis activation and SSCT fibroblast activity. Both mechanical and molecular biology approaches were used to examine differences in SSCT fibroblast behavior and collagen interactions between cells from normal tissue and cells from idiopathic CTS tissue, with and without steroid, i.e., Triamcinolone Acetonide (TA), treatment. We chose TA because it is commonly injected clinically to treat CTS [16, 18].

## Methods

### Study design

We used a gel contraction model as a measure of SSCT fibroblast activation, based on the rate of collagen gel contraction and the change of the contracted gel’s mechanical and temporal contraction properties as previously described [19]. Molecular assays of gene expression and an assay of collagen degradation products in the conditioned media were used to investigate the molecular response to TA treatment on TGF-β regulation and various molecular markers associated with CTS.

### SSCT fibroblast preparation

SSCT tissue was harvested with our Institutional Review Board’s approval. Tissues were harvested from the operated hands of 5 patients with idiopathic CTS, 3 female and 2 male, with a mean age of 58.4 (SD 4.8) years. Tissue was also harvested from the hands of same number fresh cadavers (within 12 h post mortem) of individuals with no history of CTS, with a mean age of 58.6 (SD 6.9). Patients and cadavers were excluded if they had a history of major trauma, prior steroid injection or surgery for CTS, or hand/wrist pathology, including known tumors/deformities, arthritis or metabolic disorders.

The tissue was harvested with 10x5x1 mm in size as needed from patients undergoing open carpal tunnel release and minced in minimal essential medium (MEM) with Earle’s salts (GIBCO, Grand Island, NY) modified with 10% fetal bovine serum (GIBCO) and 1% Antibiotic-Antimycotic (GIBCO). Cells were cultured in 10-cm–diameter culture dishes and grown in the same media and supplements at 37 °C, in a 5% CO_2_ humidified atmosphere, with the media changed every 3 days. The SSCT cells were passaged at 90% confluence, and passage 4 cells were used in all experiments.

### Cell-seeded collagen gel preparation

Collagen gels were prepared as previously reported [19]. Briefly, a 1.0 mg/ml collagen/modified MEM sterile solution was made from 1.88 ml Vitrogen type I bovine dermal collagen (Cohesion Technologies, Palo Alto, CA), 1.2 ml 5X-modified MEM and 2.92 ml distilled water and maintained at pH 7.4 ± 0.2 on ice. SSCT cells were seeded at 1.0 × 10^6^ cells/ml in a 0.5 mg/ml collagen/modified MEM solution and mixed into the collagen solution at room temperature. A small sterile cloning ring (8-mm outer diameter, 8-mm height) was attached to the center of each of four 3.5-cm Petri dishes with sterile vacuum grease (Dow Corning Corporation, Midland, MI). Then, the area between the ring and the outer part of each dish was filled with a 2-ml cell-seeded collagen solution and was gelled at 37 °C in a 5% CO_2_ humidified incubator for 1 h. During gelation, two media with modified MEM were prepared: one was supplemented with 10 μM TA (Sigma-Aldrich, St. Louis, MO), and the other was a dimethyl sulfoxide (Sigma-Aldrich) vehicle media. To facilitate gel contraction, a scalpel was used to release the gelled solution from the dish’s wall after 1 h of gelation. For each sample, four plates were prepared with 2 ml of media, two with TA-supplemented and two with vehicle media. All experiments were cultured at 37 °C in a 5% CO_2_ humidified incubator with the media changed every 48 h as per treatment protocol.

### Quantification and mechanical testing

Every 4 h for 3 days, the contracting gels were photographed and the areas calculated following previously described procedures [19, 20]. The gel contraction rate was determined by the change over time of the gel contraction percentage (the ratio of the current gel surface area over the initial area). Complete gel contraction was determined when gel contracted to less than 5% of original size. The decay time constant (*B*), which is directly proportional to the gel contraction rate, was calculated following an established equation (Eq. 1) [19], in which the area data at each time point were modeled with linear regression and optimization to fit an exponential decay function of time. *A*_*0*_ is the initial area (*t = 0)*; *B* is the decay time constant; and *C* is the non-zero asymptote as *t → ∞*.

1 $$ A(t)={A}_0{e}^{- Bt}+C $$

At the end of day 3, the fully contracted gel ring was taken from the dish. A customized mechanical testing system [19] was used to perform a uniaxial tensile test under displacement control at a 0.5 mm/sec distraction rate to determine stiffness and maximal tensile strength. The contracted gel ring was looped around a set of two hooks on the testing machine, which recorded force and displacement data at a sample rate of 10 Hz. The ring was submerged in a room-temperature phosphate-buffered saline (GIBCO) solution to keep the ring moist throughout the test.

### PCR Array analyses

The contracted gel was homogenized with 1 mL of Trizol reagent (Invitrogen Life Technologies, Grand Island, NY). Total RNA was isolated as previously reported [21]. cDNA was synthesized from equal quantities of total RNA with an iScriptTM cDNA Synthesis Kit (Bio-Rad Laboratories, Inc., Hercules, CA). Gene expressions was measured using Human Fibrosis PCR arrays and Cell Motility PCR arrays (PAHS-120Z and PAHS-128Z, respectively, of SA Biosciences, Frederick, MD) according to the manufacturer’s instructions. Fold changes were calculated with a log 2 scale between patient and control tissue, with and without TA treatment. Gene expression analysis was completed using SA Biosciences RT^2^ Profiler™ PCR Array Data Analysis software from The GeneGlobe Data Analysis Center of QIAGEN (https://www.qiagen.com/us/geneglobe).

### Quantitative real-time PCR

In order to confirm array results, select regulated genes from the arrays were analyzed with qRT-PCR (Table 1). Genes were analyzed in triplicate using the original cDNA, as previously described [22], and values were normalized with TATA binding protein (TBP) as a housekeeping gene. Primers were designed using Primer3web version 4.0.0 software (http://bioinfo.ut.ee/primer3) and purchased from Integrated DNA Technologies (Coralville, IA).

**Table 1:** Primers used in RT-PCR

Gene	Reference	Forward	Reverse
TGF-β1	NM_000660	GTGGAAACCCACAACGAAAT	CGGAGCTCTGATGTGTTGAA
COL1A2	NM_000089	TCCAAAGGAGAGAGCGGTAA	CAGATCCAGCTTCCCCATTA
COL3A1	NM_000090	CCAGGAGCTAACGGTCTCAG	CAGGGTTTCCATCTCTTCCA
VEGFA	NM_003376	AGGCCAGCACATAGGAGAGA	TTTCTTGCGCTTTCGTTTTT
TBP	NM_003194	GGTTTGCTGCGGTAATCATGA	CTCCTGTGCACACCATTTTCC

### Collagen degradation assessment

Conditioned media was collected after the first 48 h and centrifuged at 1500 RPM for 5 min, and the supernatant was collected and stored at − 80 °C. Conditioned media was warmed to room temperature, and CrossLaps® for Culture ELISA (Immunodiagnostic Systems, Inc., Fountain Hills, AZ) was used to determine the degradation marker, C-terminal telopeptide of type I collagen, from the breakdown of the contracted cell-seeded gel according to the manufacturer’s instructions. Briefly, preparation a two-fold dilution row of the standards, pre-dilution of test specimens with standard diluent and preparation of antibody solution were performed before the test. Then each of standards, control and samples were pipetted into appropriate wells to mix with antibody solution and incubated at room temperature for 2 h. Wash with diluted washing buffer for 5 washing cycles followed by adding substrate solution into each well and incubating for 15 min at room temperature in the dark on the mixing apparatus. Finally, add stopping solution into each well followed by measuring the absorbance at 450 nm in an absorbance microplate reader.

### Statistical considerations

The effect of cell type (patient cells or control cells) and treatment type (with TA-supplemented or vehicle media) on the outcomes of decay time constant, tensile strength, stiffness and collagen degradation concentration was analyzed. A total of 10 samples were created from patient and control cells individually (five with TA-supplemented and five with vehicle media for each cell type). Duplicate contraction tests were performed with each specimen, and the averages were used in the analyses, which were conducted using two-factor analysis of variance in a generalized linear model framework utilizing generalized estimating equations (GEE) to account for the correlation between samples. Because significant interactions were observed between cell type and treatment type in the main analysis, additional analyses were performed by generating separate one-factor models to analyze TA’s effect separately for patient and control cells. All statistical tests were two-sided and *p*-values less than 0.05 were considered significant.

## Results

### Mechanical testing of gel contraction

**Fig. 1:**
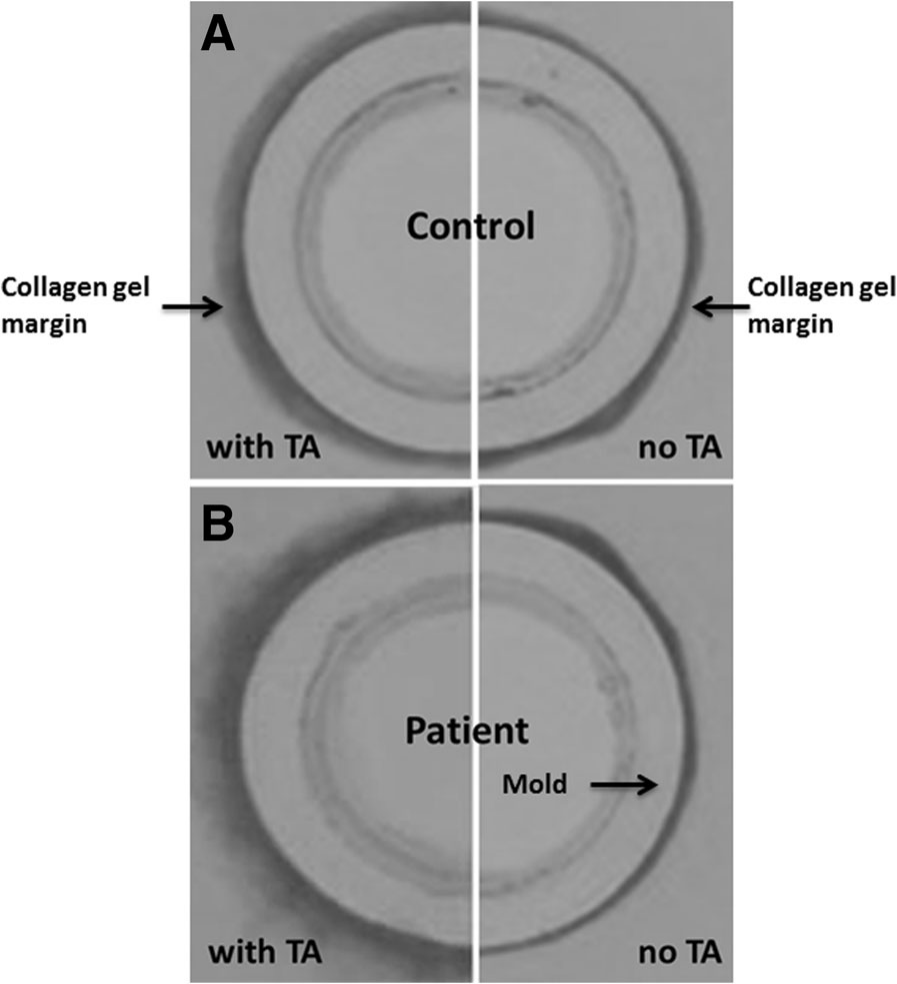
TA treatment resulted in poorly defined margin formation regardless of cell type. **a** control, **b** patient

Morphologically, in both the control and patient cell types, gels with TA-supplemented media had rough margins after full contraction (Fig. 1). The outcomes organized by factor and by group are shown in Tables 2 and 3, respectively. Regarding the main effects by factor, gels with TA-supplemented media had a significantly higher contraction rate, tensile strength and stiffness than those with vehicle media. Gels seeded with patient cells had a higher contraction rate and stiffness than those seeded with control cells, but no significant difference was observed respecting tensile strength.

**Table 2:** Summary of the analysis of the main effects on mechanical testing

Outcome	Factor	Level	Mean (SD)	*p*-value
Decay Time Constant (days-1)	Treatment Type	TA	2.9 (1.0)	< 0.001^*^
		No TA	2.2 (0.7)	
	Cell Type	Cadaver (control)	2.5 (1.1)	< 0.001^*^
		CTS patient	2.6 (0.7)	
Tensile Strength (mN)	Treatment Type	TA	7.0 (1.2)	< 0.001^*^
		No TA	5.8 (1.3)	
	Cell Type	Cadaver (control)	6.6 (1.4)	0.921
		CTS patient	6.1 (1.3)	
Stiffness (mN/mm)	Treatment Type	TA	1.1 (0.4)	< 0.001^*^
		No TA	0.7 (0.3)	
	Cell Type	Cadaver (control)	0.8 (0.3)	0.004^*^
		CTS patient	1.0 (0.4)	

* = Statistically significant

**Table 3:** Summary of analysis of the effect of TA, separately by gel type

Outcome	Cell Type	TA	Mean (SD)	*p*-value
Decay Time Constant (days^−1^)	Cadaver (control)	No TA	1.9 (0.8)	< 0.001^*^
		TA	3.0(1.2)	
	CTS patient	No TA	2.5 (0.6)	0.225
		TA	2.7 (0.8)	
Tensile Strength (mN)	Cadaver (control)	No TA	5.8 (1.3)	< 0.001^*^
		TA	7.4 (1.0)	
	CTS patient	No TA	5.8 (1.4)	0.157
		TA	6.5 (1.2)	
Stiffness (mN/mm)	Cadaver (control)	No TA	0.6 (0.1)	< 0.001^*^
		TA	1.0 (0.3)	
	CTS patient	No TA	0.9 (0.3)	0.042^*^
		TA	1.2 (0.4)	

**Fig. 2:**
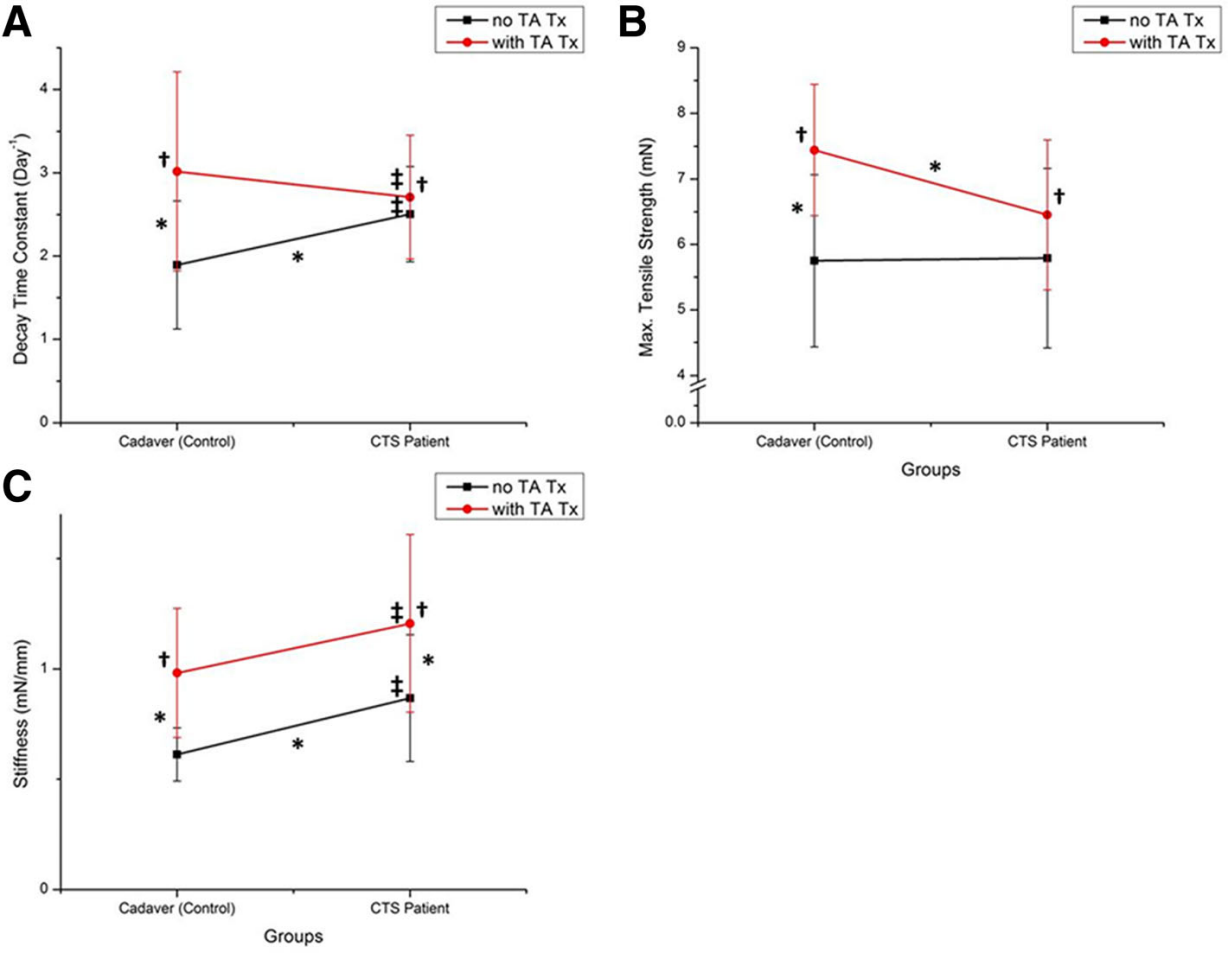
**a** Decay time constant means, **b** Tensile strength means and **c** Stiffness means for the cell type and treatment type. Bars denote mean ± SD. Two-way ANOVA showed significant effects of cell type and treatment type. †*p* < 0.05, TA treatment overall compared with vehicle control; ‡*p* < 0.05, patient overall compared with control; *p < 0.05, significant difference between groups in individual factors

Additionally, in individual group comparisons, the gels of control cells with TA-supplemented media had a higher mean contraction rate and mean tensile strength than those with vehicle media. The gels of patient cells with TA-supplemented media also had a higher mean contraction rate and mean tensile strength than those with vehicle media, but the difference was not statistically significant (Fig. 2a and b). The stiffness of the gels with TA-supplemented media was significantly higher than those with vehicle media regardless of cell type (Fig. 2c).

### PCR Array analyses of fully contracted gels

**Fig. 3:**
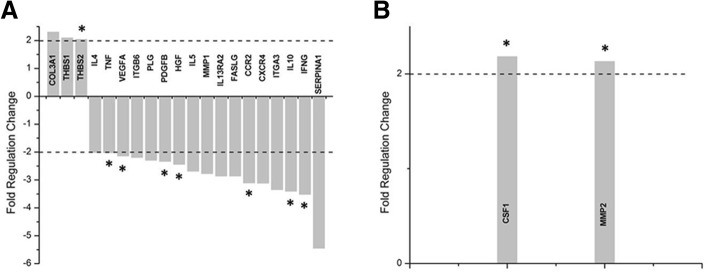
**a** Human fibrosis array and **b** Cell motility array relative fold change in genes from the patient group without TA treatment compared to the control group without TA treatment. (* indicates significant differences, *p* < 0.05)

In all comparisons, only a twofold or greater up- or down-regulation was considered to represent a changed gene expression. For the Human Fibrosis array (Additional file 1: Table S1), in the gels of patient cells with vehicle media compared to those of control cells with vehicle media, 3 genes were upregulated, e.g., collagen (COL) 3A1, and thrombospondins (THBS) 1 and 2, and 17 were down-regulated, e.g., platelet-derived growth factor (PDGF), vascular endothelial growth factor (VEGF), integrins (ITG) A3 and B6, and matrix metallopeptidase (MMP) 1 (Fig. 3a). In the Cell Motility array (Additional file 2: Table S2), 2 genes were upregulated in the gels of patient cells with vehicle media compared to those of control cells with vehicle media (Fig. 3b). But no down-regulated genes were found.

**Fig. 4:**
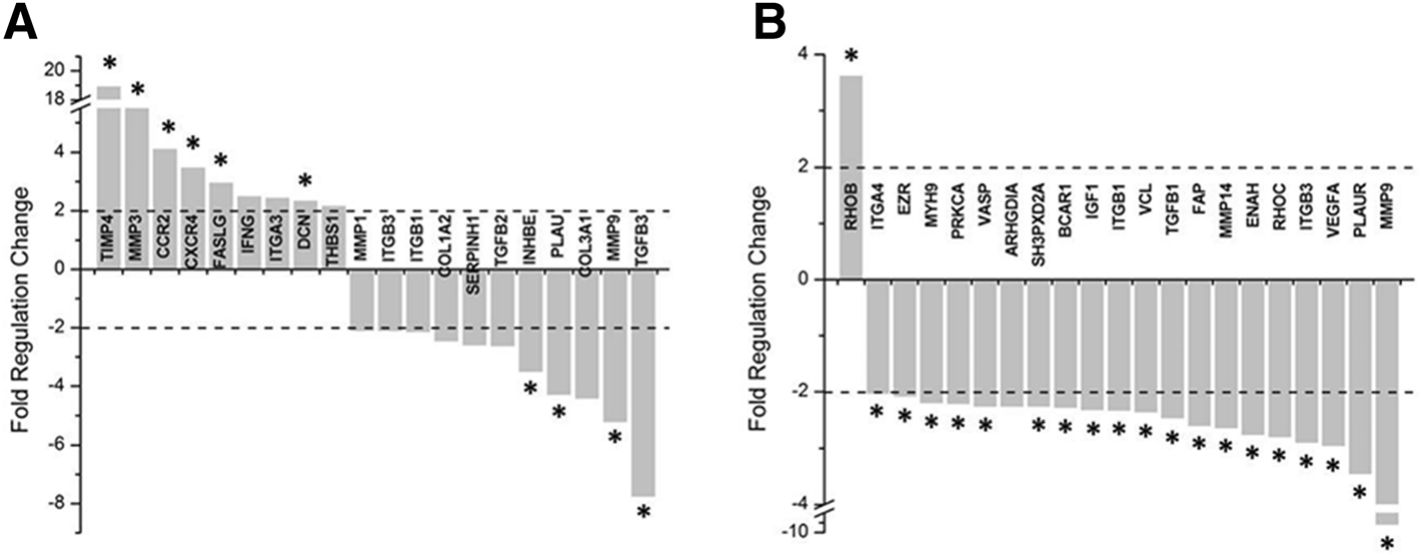
**a** Human fibrosis array and **b** Cell motility array relative fold change in genes from the patient group with TA treatment compared to the patient group without TA treatment. (* indicates significant differences, *p* < 0.05)

Regarding TA’s effect on gel contraction, in the gels of patient cells with TA-supplemented compared to those with vehicle media, 9 genes in the Human Fibrosis array were upregulated, e.g., tissue inhibitors of metalloproteinase (TIMP) 4, MMP 3 and THBS 1, and 11 were down-regulated, e.g., TGF-β 2 and 3, ITG B1 and B3, COL 3A1 and 1A2, and MMP 1 and 9 (Additional file 3: Table S3 and Fig. 4a). In the Cell Motility array (Additional file 4: Table S4), 1 gene was upregulated in the gels of patient cells with TA-supplemented compared to patient cells with vehicle media, and 20 were down-regulated (Fig. 4b).

### Quantitative real-time PCR

**Fig. 5:**
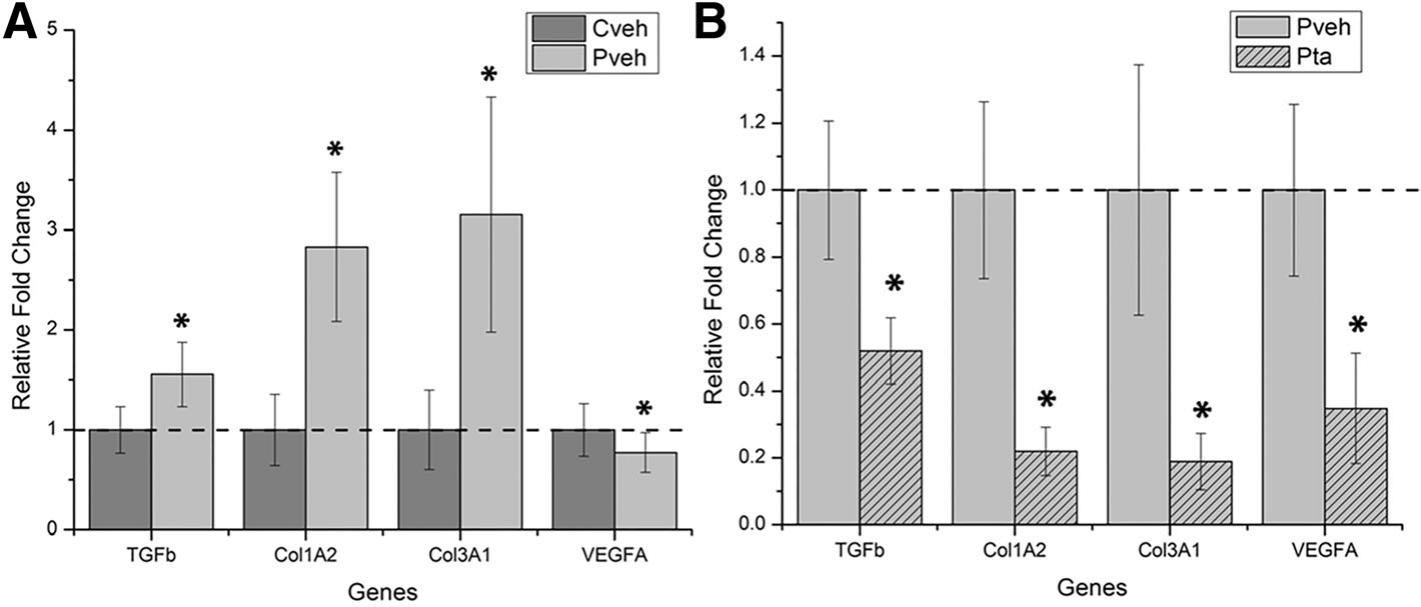
Confirmation of the arrays’ gene expressions was analyzed by quantitative RT-PCR between **a** the untreated patient group (Cveh) and the untreated control group (Pveh) (results are normalized to the untreated control group); **b** the treated patient group (Pta) and the untreated patient group (results are normalized to the untreated patient group). (* indicates significant differences, *p* < 0.05)

To confirm the microarray data, select genes that expressed twofold or greater changes in the arrays and were related to the TGF-β signaling were analyzed with quantitative Real-Time PCR (RT-PCR). All gene expressions were consistent with array results. In the comparison of patient and control cells in vehicle media, TGF-β1, COL1A2 and COL3A1 were all significantly upregulated, and VEGFA was down-regulated (Fig. 5a). Similarly, patient cells treated with TA all showed significant decreases in TGF-β1, COL1A2, COL3A1 and VEGFA as was found in the arrays (Fig. 5b).

### Collagen degradation assessment by immunoassay (ELISA)

No significant difference in collagen fragment concentration was observed between treatment types or cell types, or in individual group comparisons.

## Discussion

CTS is typified by non-inflammatory SSCT fibrosis with median nerve compression [8, 9]. Surgical decompression is the most effective therapy, but steroid injection has been the main non-operative therapy. TA and other anti-inflammatory steroids are used to treat orthopedic conditions such as tendinopathy, neuropathy, repetitive motion injuries and inflammatory symptoms and reduce pain, inflammation and limitations in mobility [23, 24]. However, the mechanism of steroid therapy is not yet completely understood. Cell-seeded gel contraction—the transformation of a collagen lattice caused by cellular action—is a useful quantitative model and has been used to study fibroblast proliferation and reorganization of structural proteins in the extracellular matrix (ECM) and the effects of TGF-β signaling on these processes [25, 26]. In this study, we sought to characterize changes in both SSCT fibroblast behavior and fibroblasts’ interaction with collagen seeded with cells from normal tissue and idiopathic CTS tissue individually, with and without TA treatment, to investigate the effect of steroid treatment on SSCT fibroblast gene expression and function in a gel ring contraction model.

The results of the mechanical property test of gels with vehicle media (Fig. 2) support those of our previous study comparing patient and control cells, although the difference in tensile strength was not significant [19]. Regarding TA treatment, the effect of blurring the margins was greater in gels seeded with patient cells (Fig. 1), suggesting that TA had a greater effect on these cells and may have impeded their ability to contract the gel. While the mechanism of action of TA when injected to treat CTS is unknown, this finding suggests that TA may somehow interfere with the progress of synovial fibrosis that is commonly seen in CTS patients. Additionally, TA treatment significantly increased gel contraction rate, tensile strength and stiffness in control cells but only stiffness in patient cells (Fig. 2), suggesting that TA has a different effect in normal and CTS-affected cells. Specifically, TA treatment seems to involve impeding collagen synthesis and reorganizing molecular cross-linking in collagen, resulting in increased tissue stiffness and tensile strength, which is consistent with previous studies [17, 27, 28].

In this study, the changes in fibrotic and ECM gene expression in gels seeded with patient cells compared to those seeded with control cells (Fig. 3) were consistent with those in previous studies, indicating that fibrotic pathways are important mediators in this model and supporting the common histopathological observation that the fibrosis seen in CTS is not associated with inflammation [8, 9, 21].

Regarding TA treatment of gels seeded with patient cells (Fig. 4), TA inhibited the expression of TGF-β–related fibrotic and ECM genes, such as collagens, TGF-βs and MMPs, and promoted expression of remodeling proteinase genes, including TIMP and thrombospondin. These results were consistent with those of previous studies, which showed that TA modulates TGF-β’s transcription and translation and in turn ECM composition [27, 29]. One mechanism of action is the steroids’ inhibition of MMP transcription, which may negatively impact collagen synthesis by suppressing protein binding activity [30, 31]. In this study, some integrins (Additional file 1: Table S1), such as ITGB1 and ITGB3, were upregulated in the gels of patient cells with vehicle media compared to those of control cells with vehicle media, although the change was less than twofold. However, in the patient cells with TA treatment, integrins were down-regulated twofold compared to those with vehicle media (Fig. 4). These results are consistent with those of previous studies, in which dexamethasone treatment of human-derived tendon cells decreased β1-integrin synthesis and reduced type I collagen production [32]. Integrins mediate translational signaling from the adjacent ECM to change cell behaviors such as adhesion, migration and proliferation to modulate cell-ECM and cell-cell adhesion, resulting in fibrosis [32, 33]. Integrins can regulate canonical and non-canonical TGF-β fibrotic signaling processes (such as systemic sclerosis, pulmonary fibrosis and cancer). Additionally, TGF-β regulates integrins to modulate cell behaviors, such as migration and adhesion [34, 35]. The cytokines VEGF and PDGF are additional known mediators of fibrosis both separately and in conjunction with TGF-β. However, in our study, PDGF and VEGF expressions were suppressed in the patient groups regardless of treatment type (Figs. 3 and 4). Thus, TA does not seem to affect these pro-fibrotic pathways. Reducing fibrosis via these other pathways may be a promising avenue for further research into medical treatments of CTS.

To understand how the untreated and TA-treated fibroblasts modulate and regulate the adjacent ECM in our gel contraction model, we examined whether collagen breakdown was a mechanism involved in the gel contraction in both control and treated conditions. Many studies have demonstrated that collagen breakdown is normally a part of tissue regeneration and that corticosteroids inhibit the degradation of mature collagen by suppressing the collagen breakdown [36, 37]. There was no significant difference in the products of collagen breakdown between gel types or treatment types, indicating that collagen breakdown is not the likely mechanism of this cell-seeded gel contraction model.

There are some limitations in this study. First, despite the gender and age match between the patient and control cells, the sample size was small. However, we were able to see significant cell and treatment interactions. Second, the phenomenon of the rough edge (margin) of contracted gel was found in all TA treatment groups regardless of cell type. In order to quantify and compare the rough edge we measured a solidly visible edge as the contraction edge to quantify the contraction rate. Third, we investigated only canonical TGF-β fibrotic signaling processes. In the future, we plan to investigate and compare the quality and arrangement, and the changes of inter−/intra-collagen fibers after contraction between treated and non-treated groups using confocal microscope and scanning electron microscope respectively. Moreover, we plan to study other possible signaling processes related to fibrosis. However, researchers may be able to use the current model to understand the dynamics of SSCT pathogenesis and treatment more clearly find ways to block signaling pathways or test new therapies.

## Conclusion

In conclusion, our results in collagen gel contraction were consistent with those of previous studies: there was an increased cell-seeded gel contraction rate, stiffness and tensile strength in gels with media of patient cells compared to those of control cells. We also found that the TA-treated groups had poorly defined margins after contraction in both cell types (though this was more pronounced in the patient groups), which suggests that TA affected gel structural integrity. Fibrotic genes and ECM regulators, such as TGF-β, collagens and integrins, were upregulated in the gels of patient cells with vehicle media compared to the gels of control cells with vehicle media. Additionally, in gels with TA-supplemented cells, the patient cell groups had increased expression of remodeling proteinases compared to the control cell groups, indicating that TA modulates TGF-β signaling and affects ECM composition. Furthermore, with TA treatment, patient cells’ fibrotic genes and ECM regulators, such as TGF-β, collagens and integrins, were down-regulated, indicating that TA may be working in part by decreasing an overall fibrotic gene expression. This study shows that steroids affect cell regulation on gel structural integrity and regulate fibrotic gene expression in patient cells and may affect CTS by modulating cellular function. This may explain the reported decreases in CTS symptoms for some patients treated with steroids. Further study of these identified genes and pathways may help researchers discover new and more effective therapeutic targets for CTS treatment.

## Additional files

Additional file 1: **Table S1.** Gene fold changes of the CTS group without TA treatment compared to the control group without TA treatment in Human Fibrosis Array (XLS 84 kb)

Additional file 2: **Table S2.** Gene fold changes of the CTS group without TA treatment compared to the control group without TA treatment in Cell Motility Array. (XLS 84 kb)

Additional file 3: **Table S3.** Gene fold changes of the CTS group with TA treatment compared to the CTS group without TA treatment in Human fibrosis array. (XLS 84 kb)

Additional file 4: **Table S4.** Gene fold changes of the CTS group with TA treatment compared to the CTS group without TA treatment in Cell Motility Array. (XLS 84 kb)
